# A Dinucleotide Deletion in *CD24* Confers Protection against Autoimmune Diseases

**DOI:** 10.1371/journal.pgen.0030049

**Published:** 2007-04-06

**Authors:** Lizhong Wang, Shili Lin, Kottil W Rammohan, Zhenqiu Liu, Jin-qing Liu, Run-hua Liu, Nikki Guinther, Judy Lima, Qunmin Zhou, Tony Wang, Xincheng Zheng, Dan J Birmingham, Brad H Rovin, Lee A Hebert, Yeeling Wu, D. Joanne Lynn, Glenn Cooke, C. Yung Yu, Pan Zheng, Yang Liu

**Affiliations:** 1 Division of Immunotherapy, Department of Surgery, Comprehensive Cancer Center, University of Michigan, Ann Arbor, Michigan, United States of America; 2 Program of Molecular Mechanisms of Disease, University of Michigan, Ann Arbor, Michigan, United States of America; 3 Department of Statistics, The Ohio State University, Columbus, Ohio, United States of America; 4 Department of Neurology, The Ohio State University, Columbus, Ohio, United States of America; 5 Department of Pathology, The Ohio State University, Columbus, Ohio, United States of America; 6 OncoImmune, Columbus, Ohio, United States of America; 7 Department of Internal Medicine, The Ohio State University, Columbus, Ohio, United States of America; 8 Department of Pediatrics, The Ohio State University, Columbus, Ohio, United States of America; Baylor College of Medicine, United States of America

## Abstract

It is generally believed that susceptibility to both organ-specific and systemic autoimmune diseases is under polygenic control. Although multiple genes have been implicated in each type of autoimmune disease, few are known to have a significant impact on both. Here, we investigated the significance of polymorphisms in the human gene *CD24* and the susceptibility to multiple sclerosis (MS) and systemic lupus erythematosus (SLE). We used cases/control studies to determine the association between *CD24* polymorphism and the risk of MS and SLE. In addition, we also considered transmission disequilibrium tests using family data from two cohorts consisting of a total of 150 pedigrees of MS families and 187 pedigrees of SLE families. Our analyses revealed that a dinucleotide deletion at position 1527∼1528 (P1527*^del^*) from the *CD24* mRNA translation start site is associated with a significantly reduced risk (odds ratio = 0.54 with 95% confidence interval = 0.34–0.82) and delayed progression (*p* = 0.0188) of MS. Among the SLE cohort, we found a similar reduction of risk with the same polymorphism (odds ratio = 0.38, confidence interval = 0.22–0.62). More importantly, using 150 pedigrees of MS families from two independent cohorts and the TRANSMIT software, we found that the P1527*^del^* allele was preferentially transmitted to unaffected individuals (*p* = 0.002). Likewise, an analysis of 187 SLE families revealed the dinucleotide-deleted allele was preferentially transmitted to unaffected individuals (*p* = 0.002). The mRNA levels for the dinucleotide-deletion allele were 2.5-fold less than that of the wild-type allele. The dinucleotide deletion significantly reduced the stability of *CD24* mRNA. Our results demonstrate that a destabilizing dinucleotide deletion in the 3′ UTR of *CD24* mRNA conveys significant protection against both MS and SLE.

## Author Summary

When an individual's immune system attacks self tissues or organs, he/she develops autoimmune diseases. Although it is well established that multiple genes control susceptibility to autoimmune diseases, most of the genes remain unidentified. In addition, although different autoimmune diseases have a common immunological basis, a very small number of genes have been identified that affect multiple autoimmune diseases. Here we show that a variation in *CD24* is a likely genetic factor for the risk and progression of two types of autoimmune diseases, including multiple sclerosis (MS), an organ-specific autoimmune disease affecting the central nervous system, and systemic lupus erythematosus, a systemic autoimmune disease. Our data indicated that if an individual's *CD24* gene has a specific two-nucleotide deletion in the noncoding region of *CD24* mRNA, his/her risk of developing MS or SLE is reduced by 2- to 3-fold. As a group, MS patients with the two-nucleotide deletion will likely have a slower disease progression. Biochemical analysis indicated that the deletion leads to rapid decay of *CD24* mRNA, which should result in reduced synthesis of the CD24 protein. Our data may be useful for the treatment and diagnosis of autoimmune diseases.

## Introduction

Multiple sclerosis (MS) is a chronic, inflammatory neurodegenerative disease of the central nervous system of unknown etiology. There is evidence to support the hypothesis that MS is an autoimmune process modulated by both genetic and environmental factors [1–6]. An increased risk of MS among MS relatives has been found in numerous prospective epidemiological studies [2,4,7]. Twin studies from different populations consistently indicate that a monozygotic twin has a 5- to 6-fold higher risk of MS than a dizygotic twin [1,2,8]. Collectively, these findings would implicate that, at least in part, the risk for developing this disorder and possibly its progression are mediated by multiple genetic factors. Several whole-genome screens were performed in MS affected families. These studies confirmed the association of MS with the *HLA* class II *DR2* haplotype (HLA-DRB1*1501-DQA1*0102-DQB1*0602), but failed to confirm other major putative loci in MS [9–11].

Systemic lupus erythematosus (SLE) is a classic systemic autoimmune disease with diverse clinical symptoms, including fatigue, joint pain and swelling, skin rashes, and chest pain. Severe SLE complications include nephritis, central nervous system vasculitis, pulmonary hypertension, interstitial lung disease, and stroke. Whole-genome scans have revealed multiple chromosomal regions [12–17]. However, the identity of most susceptibility genes are unknown [18].

CD24 is a glycosylphosphatidylinositol-anchored cell surface protein with expression in a variety of cell types that can participate in the pathogenesis of MS and SLE, including activated T cells [19,20], B cells [21], macrophages [22], and dendritic cells [23]. *CD24,* as a candidate locus [10], was shown to be essential for the induction of experimental autoimmune encephalomyelitis (EAE) in mice [24]. Interestingly, CD24 controls a checkpoint of EAE pathogenesis after the autoreactive T cells are produced [24]. Recently, we showed that CD24 is essential for local clonal expansion and persistence of T cells after their migration into the central nervous system and that expression of CD24 on either hematopoietic cells or nonhematopoietic antigen-presenting cells in the recipient is sufficient to confer susceptibility to EAE [25]. These findings suggest that CD24 is essential for susceptibility to EAE.

Human *CD24* (CD24) mRNA has a 0.24-kb ORF and a 1.8-kb 3′ UTR. A CT single nucleotide polymorphism (SNP) at position 170 from the *CD24* translation start site (P170) in the CD24 putative cleavage site for the glycosylphosphatidylinositol anchor (−1 position) [26] results in the nonconservative replacement of alanine with valine. The P170*^TT^* genotype expressed higher cell-surface CD24 than the P170*^CT^* or P170*^CC^* genotypes, which had an increased risk and more rapid progression of MS [27]. Thus, the CD24 SNP may influence MS pathogenesis by affecting the expression of CD24. The potential contribution of CD24 to SLE has not been studied. However, since CD24 has emerged as a major checkpoint of homeostatic proliferation in lymphopenic hosts [28,29], and since leucopenia is a defining feature of SLE [30], it would be of great interest to evaluate whether CD24 polymorphism may affect susceptibility to SLE.

Interestingly, the long 3′ UTR of mouse *Cd24* mRNA plays an important role in controlling CD24 expression [31]. Two *cis* elements of mouse *Cd24* mRNA, a negative and a positive *cis* element, regulate the stability of mouse *Cd24* mRNA expression and determine cell-surface CD24 expression [31]. Our sequencing analysis of the 3′ UTR of *CD24* revealed four polymorphisms in the Ohio population. Considering the importance of CD24 in the development and progression of MS, we investigated the association of the *CD24* polymorphisms at the 3′ UTR with the susceptibility to both organ-specific and systemic autoimmune diseases. Our study revealed a dinucleotide deletion in the 3′ UTR of human *CD24* that confers significant protection against the risk and progression of MS and the risk of SLE by destabilizing *CD24* mRNA.

## Results

### *CD24* Chromosomal Location and Polymorphisms in the 3′ UTR

*CD24* has been identified as an autosomal gene located in Chromosome 6q21 [32], with intronless pseudogenes in Chromosomes 1, 15, and Y. In addition to a lack of introns, it has been reported that Chromosome Y DNA sequence differs from *CD24* cDNA at 23 positions, with two changes in the coding regions and the remaining ones scattered through the 1.6-kb cDNA region [32]. The *CD24* gene sequence, as assembled by Celera, is presented in Figure S1. However, a recent update in the National Center for Biotechnology Information (NCBI) database placed *CD24* on Chromosome Y with partial intron 1 sequence and exon 2 identical to the cDNA except for eight changes in the region corresponding to the 3′ end of the cDNA. We used PCR primers antisense to a portion of the intronic sequence and the 3′ end of the *CD24* mRNA to amplify from genomic DNA, using as templates genomic DNA from eight unrelated normal individuals (four males and four females). Since the primers would amplify both the Chromosome 6 sequence and the putative Chromosome Y sequence (Figure S2), we sequenced five clones from each of the eight individuals in order to determine which annotation is correct. We found that none of the 40 sequences matched the putative Chromosome Y sequence, regardless of the sex of the donor, and all sequences matched the intron 1 and exon 2 sequence of the *CD24* gene as located on Chromosome 6 [32] (Figure S2). These results indicated that the putative Chromosome Y sequence is likely incorrect and that the PCR primer pair amplifies the autosomal *CD24* gene.

**Figure 1:**
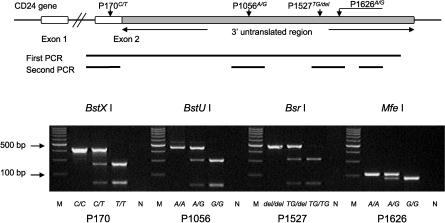
Diagram of the *CD24* Gene and Genotyping of Four Polymorphic Sites by PCR-RFLP The upper panel shows the relative position of the 3′-UTR (gray box) and the two codon regions (white boxes). Intron 1 is represented as a separate line; however, the large intron 1 is not fully represented in the figure. The relative position of each polymorphism found in the study is shown by a downward arrow. The position of the nested-PCR primers is also shown. Lower panel shows genotyping by PCR-RFLP analysis using BstX I, BstU I, Bsr I, and Mfe I restriction enzymes for P170, P1056, P1527, and P1626, respectively. The genotype of each pattern is indicated at the bottom of each lane. M: molecular size marker (lane 1, 6, 11, and 16). Numbers on the left side are the size of a standard DNA marker (bp). N: negative control (lane 5, 10, 15, and 20).

Analysis of five clones from each of the eight individuals also revealed four SNPs, three of which were reported in the NCBI database (P1056 A/G, P1527 TG/del, and P1626 A/G). As shown in Figure 1, following an anchored PCR designed to eliminate the contribution of intronless *CD24* pseudogenes [32], these three polymorphic sites could be identified by restriction enzyme digestion of individual PCR products. The accuracy of the PCR-restriction length fragment polymorphism (RFLP)was confirmed by sequencing the PCR products from 32 individuals. The PCR-RFLP analysis was therefore adopted for genotyping. The genotype distributions of these polymorphisms did not deviate from the Hardy-Weinberg equilibrium (Table S1). Moreover, the genotype distributions are essentially the same among males and females among the large set of samples tested (Table S1). We also used Merlin software (http://www.sph.umich.edu/csg/abecasis/Merlin) to detect potential genotyping errors [33]. No Mendelian inconsistency and obligatory double recombination were found. Taken together, these data ruled out the possibility that the Chromosome Y locus contributes to the data presented in this study and confirmed the accuracy of the genotypes presented.

### Case-Control Studies on 3′-UTR Polymorphisms and the Risk of MS

We examined the association of the *CD24* polymorphisms in the 3′ UTR with MS using DNA from independent Caucasian participants with MS and race-, age- and gender-matched controls from Central Ohio (Table 1). A summary of the *CD24* allele and genotype analyses of the MS patients compared with controls is shown in Table 2. A significant difference in the allelic frequencies between the MS patients and the controls was found for P1527 (*p* = 0.006), but not for P1056 or P1626.

**Table 1:**
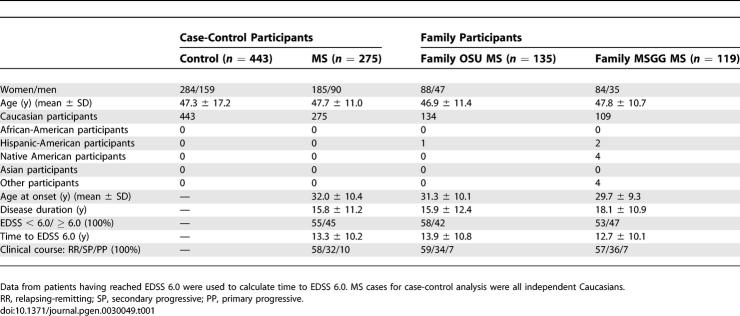
Characteristics of MS Patients and Controls

**Table 2:**
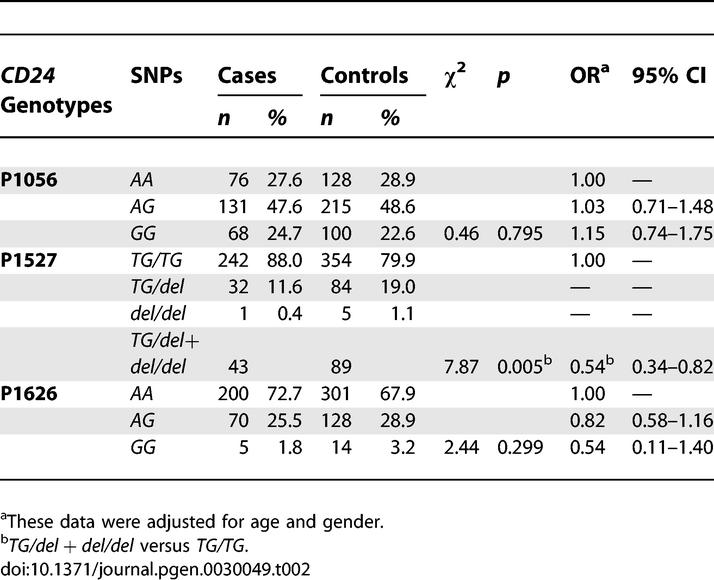
*CD24* Genotype Frequencies for All MS Participants and Controls

Remarkably, an approximately 2-fold decrease in the risk for MS was found in participants with the P1527*^TG/del^* or P1527*^del/del^* genotypes compared with the P1527*^TG/TG^* genotype. These data suggest that the dinucleotide deletion may confer protection against MS risk.

### Family-Based Tests for the Association between *CD24* Polymorphisms and MS

Since the above case-control results could potentially be due to population admixture, even though we have restricted the analysis to only the Caucasian samples, we also considered transmission disequilibrium tests using family data, as such a test is still valid under population admixture. We used a total of 150 pedigrees, including 49 pedigrees from the Multiple Sclerosis Genetics Group (MSGG) (with 63 informative nuclear families) and 101 from central Ohio (with 93 informative nuclear families), to determine whether the *CD24* polymorphisms are associated with MS risk. The family compositions of both cohorts are shown in Table S2. A strong association in P1527 was found using TRANSMIT (http://www-gene.cimr.cam.ac.uk/clayton/software) [34,35] (*p* = 0.002). No significant association was observed with other SNPs.

**Figure 2:**
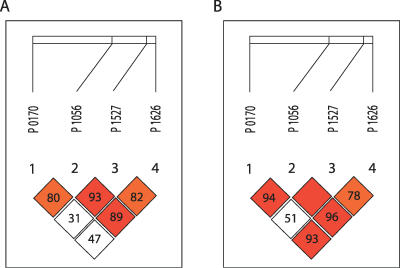
LD Analysis of Four Polymorphic Sites in the CD24 Gene The data from two cohorts of families are presented. Pairwise LD measure *r* ^2^ in the display was calculated for each of the six pairs of SNPs using data from three cohorts of families and the results are displayed in a tilted matrix. (A) Data from the OSU and MSGG families. (B) Data from the SLE families' samples from Columbus Children's Hospital. Complete LD was observed between 1056 and 1527/1626 in the SLE family sample cohort between 1056 and 1527.

Linkage disequilibrium (LD) analysis of the four SNPs using the 150 MS and 187 SLE family samples revealed a surprisingly low LD between P170 and P1527 (Figure 2A and 2B). Considering the short distance between the SNPs, it is possible that a recombination hotspot may exist in the *CD24* gene. These results, together with the fact that P1056 and P1626 are not significantly associated with MS susceptibility, suggest that P170 and P1527 are independently associated with MS risk. Such an interpretation is plausible since the allele frequencies of the SNPs are not very similar, which diminishes the power of detecting association even for a nearby SNP in high LD with the causal SNP. The significance of the origin of the participant in the association of P170 has been highlighted in recent studies by us and others [27,36,37].

### The Association of SNPs P1527 and P1626 with the Progression of MS

MS disease severity is usually measured according to the expanded disability status scale (EDSS). MS patients who have lost the ability to walk without aid have reached EDSS 6.0. For the majority of the patients, their EDSS 6.0 status was based on a follow-up visit to our center. A few of the cases were based on case history. Because this is one of the most traumatic events in a patient's life, most can recall accurately the time when their disease reached EDSS 6.0. We then tested whether the CD24 genotype affected the time span it took the patients to reach EDSS 6.0 from the day of the first symptom of MS. Clinical data from 275 independent Caucasian MS patients in the Ohio cohorts, but not those from the MSGG, were available for the survival analysis.

**Figure 3:**
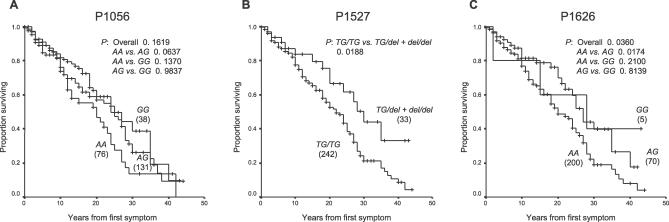
Kaplan-Meier Curves for Overall Survival by *CD24* Polymorphisms among OSU MS Patients (A) No significant difference was found in the survival rate among patients with the P1056 genotype. (B) Patients carrying the variant P1527*^del^* allele had a higher survival rate than those who had two copies of the wild-type P1527*^TG^* allele. (C) Patients with the P1626*^AA^* genotype had a lower survival rate than those with the P1626*^AG^* or P1626*^GG^* genotype. Numbers in parentheses are the size of samples for MS patients. Forty-five percent of the MS patients have reached EDSS 6.0.

The Kaplan-Meier curves provide estimates of the distribution of the time it took to reach EDSS 6.0 for patients with different genotypes. As shown in Figure 3, patients with the P1527*^TG/del^* or P1527*^del/del^* genotype had a more delayed disease progression pattern than those with the P1527*^TG/TG^* genotype (*p* = 0.0188). In addition, the patients with the P1626*^AA^* genotype also showed faster progression (*p* = 0.0105). No significant result was found in the patients with the P1056 genotype.

### Case-Control Studies on the 3′ UTR Polymorphism and the Risk of SLE

We used a Caucasian cohort of age and sex-controlled samples (Table 3) to test the potential association between *CD24* polymorphism and risk of SLE. A summary of the *CD24* allele and genotype analyses in the SLE patients against controls is shown in Table 4. A significant difference in the allelic frequencies between the SLE patients and the controls was found for P1527 (*p* = 0.00003), but not for P1056 or P1626. Remarkably, a 2.6-fold decrease in the risk for SLE was found in participants with the P1527*^TG/del^* or P1527*^del/del^* genotype compared to the P1527*^TG/TG^* genotype. These data suggest that the dinucleotide deletion may confer protection against SLE risk.

**Table 3:**
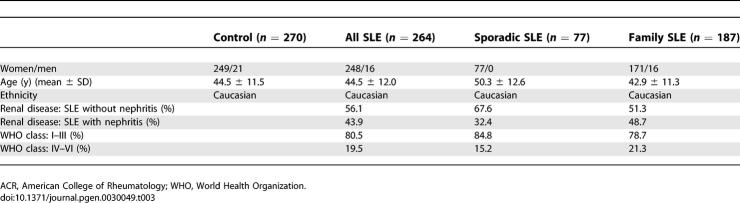
Characteristics of SLE Patients and Controls

**Table 4:**
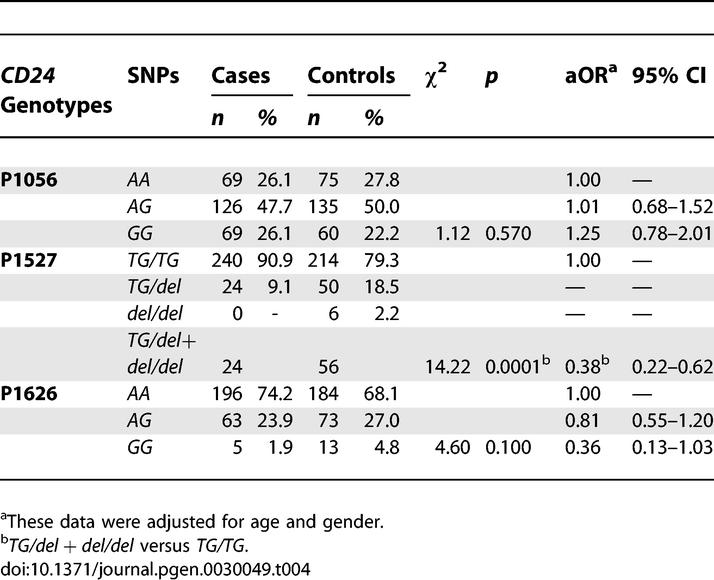
*CD24* Genotype Frequencies for All SLE Participants and Controls

### Family-Based Tests for the Association between *CD24* Polymorphisms and SLE

We used a total of 187 pedigrees to determine whether the *CD24* polymorphisms are associated with SLE risk (with 187 informative nuclear families). A strong association in P1527 was found using TRANSMIT (*p* = 0.002), but it did not show evidence for transmission disequilibrium for P170, P1056, or P1626 (*p* > 0.05). Linkage disequilibrium analysis of the four SNPs using the 187 family samples also revealed a low LD between P170 and P1527 (Figure 2B), suggesting that P1527 is independently associated with SLE risk, the same as in MS.

### Dinucleotide Deletion at P1527 Leads to an Allele-Specific Reduction of *CD24* mRNA

**Figure 4:**
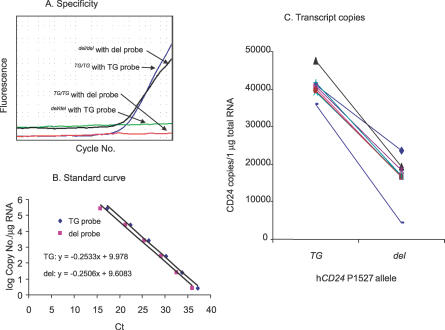
Allele-Specific Transcripts in P1527*^TG/del^* Heterozygous Individuals Total RNA was isolated from the blood of eight OSU MS patients with the P1527*^TG/del^* heterozygous genotype. The allele mRNA expression of CD24 was analyzed using TaqMan RT-PCR. (A) Specificity of the primers. DNA from patients homozygous for either *TG* or *del* at the P1527 was amplified with allele-specific primers. Note that no products were detected when the primers and the patient genotypes were mismatched. (B) Standard curve of the allele-specific amplification. The known copies of plasmid cDNA were used as templates. (C) Quantification of allele-specific *CD24* transcripts. Total RNA from eight P1527*^TG/del^* patients was amplified with allele-specific primers. The copy numbers were calculated based on the standard curves. A significant difference in the mRNA expression of *CD24* was observed between the P1527*^del^* and P1527*^TG^* alleles (*p* < 0.0001). Data shown have been repeated twice.

Since P1527 resides in the 3′ UTR, its polymorphism may affect the accumulation of its mRNA. To address whether mRNA transcribed from the P1527*^del^* allele presents a decrease in its expression levels in vivo, we established an allele-specific real-time PCR (RT-PCR) to measure the allele-specific transcripts. As shown in Figure 4A, the primers designed for the P1527*^TG^* allele detected *CD24* mRNA in the P1527*^TG/TG^*, but not in the P1527*^del/del^* individuals, and vice versa. These results demonstrate complete specificity of the primers used. In addition, the conditions used led to the amplification of *CD24* cDNA in a strictly dose-dependent fashion over six logs of magnitude (Figure 4B). We therefore used this method to measure the allele-specific expression of two *CD24* alleles in eight P1527*^TG/del^* individuals. As shown in Figure 4C, the P1527*^del^* transcripts were 2.5-fold less than the P1527*^TG^* transcripts. Since the two alleles were present in the same cells and therefore were transcribed at the same rate, our data demonstrate that the P1527 variant has a strong impact on mRNA expression of *CD24* in vivo, most likely by post-transcriptional mechanisms.

### P1527 Modulates CD24 mRNA Stability

**Figure 5:**
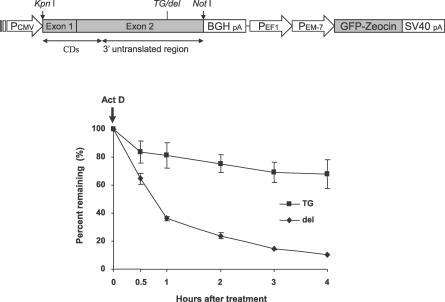
Dinucleotide Deletion at P1527 Destabilizes *CD24* mRNA The top panel depicts vector constructs used in the analysis. The *CD24* cDNA and the GFP-Zeocin have a different promoter and poly-A site cassette, respectively. Vectors with different haplotypes, CD24*^TG^* and CD24*^del^*, were used to transfect CHO cells. The lower panel shows the kinetics of mRNA degradation. The amount of *CD24* mRNA was quantified using RT-PCR. Actinomycin D treatment was administered at 48 h after transfection, and the total RNA was extracted at 0, 0.5, 1, 2, 3, and 4h after actinocmycin D treatment. The relative amount of *CD24* mRNA was calculated as the percentage of untreated mRNA. A significant difference in *CD24* mRNA degradation was observed between CD24*^TG^* and CD24*^del^* haplotypes (*p* < 0.0001, Fisher's PLSD test). Error bars represent the standard deviation of mean. Data shown have been repeated twice.

P1527 is located in the 3′ UTR that modulates mRNA stability [31]. To test if this polymorphism modulates *CD24* mRNA stability, we constructed two plasmids (pTracer CMV2-CD24*^TG^* and pTracer CMV2-CD24*^del^*; Figure 5, top panel) and transfected Chinese hamster ovary (CHO) cells with the two constructs. Starting at 48 hours after transfection, the synthesis of RNA was blocked by actinomycin D, and the half life of mRNA was measured by RT- PCR. The levels of GFP mRNA were used as internal controls for transfection efficiency.

Prior to actinomycin D treatment, there was significantly higher mRNA expression for the CD24*^TG^* cDNA in comparison to the CD24*^del^* cDNA. Using the pre-treatment mRNA levels as 100%, we measured the decay kinetics of two mRNA from two different cDNA. As shown in Figure 5 (lower panel), the decay patterns of CD24*^TG^* were significantly more gradual than those of CD24*^del^* (*p* < 0.001), and the differences in the rates of decay were significantly different at all time points starting from 0.5 h (all *p* < 0.001). In particular, mRNA from the CD24*^del^* cDNA had a half life of less than 1 h, while that derived from the CD24*^TG^* had a half life of more than 4 h. Thus, the dinucleotide deletion at the P1527 position destabilized *CD24* mRNA.

## Discussion

It is well established that polymorphisms of immune-related genes modulate host susceptibility to autoimmune diseases, including MS and SLE [27,38–42]. Historically, most studies have focused on polymorphisms that result in the replacement of amino acids [27,38,40]. More recently, substantial information has been accumulated that demonstrates that polymorphisms at the promoter and intron regions can also have a significant impact on susceptibility. These alterations modulate either RNA synthesis (transcription) or splicing [41,42]. Although it is well established that the 3′ UTR plays a major role in RNA stability, we are not aware of any study reporting that polymorphism at the 3′ UTR modulates susceptibility to autoimmune diseases by changing mRNA stability. Our data presented in this study revealed that a destabilizing dinucleotide deletion in the 3′ UTR of the CD24 gene may confer a significant protection against the risk and progression of MS and against the risk of SLE. Our conclusion is based on five lines of evidence.

First, a population study with 275 independent Caucasian MS patients and a comparable size of normal controls revealed that individuals with the deletion in at least one allele had about a 2-fold less relative risk in comparison to those without the deletion. Thus, the CD24 P1527*^del^* allele may be a protective genetic susceptibility factor for the onset of MS. This is more remarkable in light of the fact that polymorphisms at sites that were only 100–500 bp apart did not have a significant impact on the risk of MS. The strong association at P1527, but not at the nearby SNPs, suggests that the deletion was causatively related to the reduced MS susceptibility. This interpretation is consistent with the fact that the frequencies of the associated alleles at the two nearby (flanking) loci are very different from that of the protective allele. A recent study showed that the power to detect the association in such loci is diminished even when there is high linkage disequilibrium [43]. This also leads to a reasonable explanation as to why two loci in high LD are not both associated with the disease.

Second, using data from two independent cohorts of families, we also established a strong association of the CD24 P1527 polymorphism with MS. The P1527*^TG^* allele was preferentially transmitted to affected individuals. This result strongly supports the conclusion from the case-control analysis that the P1527*^del^* allele may be a protective genetic susceptibility factor for the onset of MS. Both of these results remain significant after multiple-testing adjustments. Within the Ohio State University (OSU) cohort, our previous data revealed that the P170*^T^* allele was preferentially transmitted into affected individuals among multiplex families with two or more MS patients [27]. This result continues to hold with our expanded OSU family set, although not with the MSGG cohort (data not shown). In summary, results from both of population and the family studies confirm our earlier conclusion that the *CD24* locus is a major modulator for MS risk.

Third, survival analysis revealed a significant association (even after correcting for multiple tests) of *CD24* P1527 with MS disease progression; MS patients with the P1527*^del^* allele had a significantly delayed progression. This finding further confirms that the P1527*^del^* allele is a protective genetic factor for MS. An interesting issue is whether P1527 is associated with the progression of MS because of its linkage to P170. We consider it very unlikely as our analysis of LD revealed that there is little LD between the two sites despite their close proximity to one another, perhaps due to a recombination hotspot within the *CD24* gene. Moreover, P1056, which is closer to P170, is not associated with the progression of MS. We therefore consider it likely that P170 and P1527 are independently associated with the progression of MS. Since P1626 is less than 100 bp away and shows a strong LD with P1527, it remains possible that its association with MS progression may be due to its proximity to P1527. This interpretation is favored as P1626 shows no association with MS risk. Since our analysis has now covered all known CD24 polymorphisms in the exons, it is likely that P1527, rather than other SNPs, is related to protection against autoimmune diseases.

Fourth, in addition to MS, which is an organ-specific autoimmune disease, we also observed that the *CD24* P1527*^del^* allele is preferentially transmitted to unaffected individuals in the SLE family data. It is worth noting that the SLE data should not be regarded as a replication of MS data per se. Rather, our data suggest that the protective effect of the dinucleotide deletion extends to systemic autoimmune diseases. Thus, in addition to its critical role for T-cell proliferation in the central nervous system [25], CD24 may play a role in the development of multiple autoimmune diseases.

Based on the observed data pattern and the structure of the family cohorts, we have chosen TRANSMIT soft ware to detect association between CD24 polymorphism and risk of autoimmune diseases to maximize the statistical power. However, we caution that TRANSMIT may have inflated type-I error due to its inferences of missing parental genotypes [44]. Nevertheless, we do not believe the core finding is due to type-I errors, as statistically significant association can also be find with FBAT that deletes data from families without parental information (MS dataset, *p* = 0.04; SLE data set, *p* = 0.01).

Fifth, the dinucleotide deletion reduced steady levels of *CD24* mRNA by more than 2-fold. Thus, in heterozygous patients, the mRNA from the alleles with the deletion was only 50% of that of the alleles without the deletion. This is recapitulated in transfection studies. Analysis of RNA decay kinetics revealed that the half life for the CD24 transcript with the dinucleotide deletion was at least 4-fold shorter than that of the wild-type allele. Since *CD24* was expressed at high levels among some lineages of hematopoietic cells and in the transfected CHO cells, the reduction in the steady levels may underestimate the reduction in other cell types, such as T cells, in which *CD24* is expressed at lower levels and is therefore less likely to saturate the degradation system. The low expression of *CD24* in T cells is essential for homeostatic proliferation of T cells, which has been implicated in the development of autoimmune diseases.

In summary, we demonstrated that a dinucleotide deletion at the 3′ UTR of the human *CD24* gene confers significant protection against the risk and progression of MS and the risk of SLE. These results not only provide insight into the genetic basis of MS and SLE susceptibility, but, perhaps more importantly, to our knowledge, this is the first report that shows how polymorphisms at the 3′ UTR modulate susceptibility to autoimmune diseases by regulating RNA stability. Since CD24 is a checkpoint for homeostatic proliferation of T cells [29], which is implicated in other autoimmune diseases [45], it will be of great interest to test the contribution of CD24 to the risk and progression of other autoimmune diseases.

## Materials and Methods

### MS patient samples.

All sample collection and experimentation was approved by the Institutional Review Board, and informed consents from all participants were obtained before sample collection. Some of the participants had been enrolled in the previous study [27]. Patients with definite MS, as diagnosed by K.W.R. and D.J.L. at OSUMultiple Sclerosis Center according to the McDonald criteria [46], were offered the opportunity to participate. The clinical diagnosis of MS type and the EDSS score [47] were determined by three of the authors (K.W.R., D.J.L., and N.G.). The time of disease onset and the time when a walking aid was first prescribed (EDSS 6.0) were determined retrospectively by the analysis of case records without knowing *CD24* genotype.

In the case-control study, we selected a consecutive series of 829 participants including 361 MS patients and 468 normal controls. For the case-control analysis based on participants with the same genetic background, we only used 275 independent Caucasian cases and age and gender-matched 443 Caucasian controls (Table 1). All MS patients were recruited at the OSU Medical Center between January 2000 and March 2006 and agreed to participate in this study. All donors gave written informed consent. The control participants were obtained from the American Red Cross (Columbus, Ohio) between September 1999 and January 2006 using leftover peripheral blood samples.

In the Ohio family study, 101 pedigrees of MS families were used for association analysis. Of the 346 participants from the families, 135 were MS patients and 211 were non-MS relatives. All MS patients and their unaffected family members were recruited at the OSU Medical Center, and all agreed to participate in this study. We interviewed all MS patients for family history of MS. Consenting family members with or without MS provided blood samples as well. In rare cases, when family members were at other locations, samples were obtained by local physicians or nurses and transported or mailed to our center. Ascertainment of the presence or absence of MS among the relatives was by history alone. Relatives who provided blood samples were not subjected to neurological evaluation or an MRI at our center. These participants were selected between October 2001 and May 2005.

In the MSGG family study, 321 participants from 49 pedigrees of multiplex MS families were obtained from the MSGG through the University of California San Francisco. Of the participants, 119 were MS patients and 202 were from non-MS relatives. These participants were selected between October 1997 and January 2003.

Demographics and disease characteristics of the MS patients and controls are summarized in Table 1. The sex ratio and average age of the OSU MS patients were not significantly different from those of normal controls (*p* = 0.506 in sex; *p* = 0.970 in age). In all of the OSU MS patients, as well as in each of the familial and sporadic groups, there were no significant correlations among age, age at onset, EDSS, duration of the disease, and clinical course (all *p* > 0.05). In the OSU MS patients, no information was obtained for the EDSS score in four patients and the clinical course in three patients. The group of patients with some missing phenotypic information was included in our genetic analysis to be detailed below.

The comparison of clinical and demographic features between OSU and MSGG family MS patients did not show any significant differences (*p* > 0.05). Although there was no significant difference in the ethnicity between the MS patients of the OSU and the MSGG families, the MS patients of the MSGG families were from a number of other countries besides the United States.

### SLE patient samples.

Demographics and disease characteristics of the SLE patients and controls are summarized in Table 3. A total of 264 unrelated SLE patients were consecutively recruited at the Columbus Children's Hospital and Research Institute, OSU, and followed in the Ohio SLE Study. SLE cases for case-control analysis were all independent Caucasians. Healthy race-, sex-, and age-matched participants (270) with no history of autoimmune disease were enrolled from the American Red Cross (Columbus, Ohio). The sex ratio and average age of SLE patients were not significantly different from those of normal controls (*p* = 0.435 in sex; *p* = 0.990 in age). The healthy participants were completely independent from the control participants in the MS group. Both case and control samples were collected between 1999 and 2006.

A large collection of 187 pedigrees of SLE families was obtained from the Columbus Children's Hospital and Research Institute, OSU, with predominantly one affected offspring per family. Of the 555 participants from the families, 187 were SLE patients and 368 were non-SLE relatives. Samples from both parents were available for 36% of the families, and samples from siblings were also collected where available (Table S2). In the case of single-parent families, samples were always taken from siblings. An extensive questionnaire and interview with a trained physician were completed by unaffected family members to determine the absence of SLE.

The SLE patients were diagnosed according to the classification criteria of the American College of Rheumatology [30,48]. Only those that were diagnosed as definitive SLE were included in the study. The demography and clinical data for the samples were listed in Table 3, using kidney involvement and WHO classifications for disease severity [48]. All participants were Caucasians who gave written informed consent. Approval for human study protocols was obtained from the human subjects review board at OSU and the Institutional Review Board.

### Polymorphism identification.

The genomic DNA was isolated from peripheral blood leukocytes (PBL) by using the QIAamp DNA Blood Minikit (Qiagen, http://www.qiagen.com). We searched for polymorphisms in the 3′ UTR of exon 2 using PCR and DNA sequencing, and these polymorphisms were further determined by DNA cloning and sequencing. Since several intronless *CD24* pseudogenes have been identified in the human genome [32], the functional *CD24* locus was selectively amplified by nested PCR (Figure 1). The first PCR amplification (Invitrogen http://www.invitrogen.com) was from intron 1 to the end of exon 2 by using a forward primer (5′-CTA AAG AGA ATG ACC TTG GTG GGT TGA G-3′) and a reverse primer (5′-CAC AGT AGC TTC AAA ACT GTT CGA-3′). The PCR conditions were as follows: 94 °C for 30 s, 55 °C for 30 s, and 68 °C for 2 min for 20 cycles. The predicted *CD24* PCR fragment was 2,017 bp long. The identity of the PCR products to the *CD24* gene sequence on Chromosome 6, but not the putative Chromosome Y locus sequence as well as the SNP in the region was confirmed by cloning and sequencing of the PCR products (Figure S2). The second PCR amplification (Promega, http://www.promega.com) was based on each polymorphic site using the primers as follows: a forward primer (5′-CTA AAG AGA ATG ACC TTG GTG GGT TGA G-3′) and a reverse primer (5′-GGA TTG GGT TTA GAA GAT GGG GAA A-3′) for 170 *C/T* polymorphism (P170) from the *CD24* translation start site, a forward primer (5′-GGC ATT TCC TAT CAC CTG TTT-3′) and a reverse primer (5′-AAT CTA CCC CCA GAT CCA AGC A-3′) for 1056 *A/G* polymorphism (P1056), a forward primer (5′-GCA ATT TTG CCT TCA AAA CAG-3′) and a reverse primer (5′-TTT AGG CTT AGG ACC AGG TTC-3′) for 1527∼1528 *TG/del* polymorphism (P1527), and a forward primer (5′-CAA CTA TGG ATC AGA ATA GCA ACA AT-3′) and a reverse primer (5′-GGAACATCTAAGCATCAGTGTGTG-3′) for 1626 *A/G* polymorphism (P1626). The PCR conditions were as follows: 94 °C for 30 s, 55 °C for 30 s, and 72 °C for 30 s, for 35 cycles. The PCR products were digested overnight with BstXI (50 °C) for P170, BstUI (60 °C) for P1056, BsrI (65 °C) for P1527, and MfeI (37 °C) for P1626 (New England Biolabs, http://www.neb.com) and then electrophoresed on 3.0% agarose gels (Figure 1). The genotypes were designated as *“C,” “A,” “del,”* or “*A*” when the restriction sites of BstXI, BstUI, BsrI, and MfeI were respectively absent, and as *“T,” “G,” “TG,”* or *“G”* when each restriction site was respectively present (Figure 1). The validity of the PCR-RFLP analysis was confirmed by direct sequencing of several PCR samples with each genotype.

### Molecular cloning and plasmid construction.

*CD24* cDNA was amplified from the peripheral blood leukocyte of individuals with the P1527*^TG/del^* genotype by RT-PCR (Invitrogen). The following primers were used: a forward primer (5′-ATG GGC AGA GCA ATG GTG-3′) and a reverse primer (5′-CAC AGT AGC TTC AAA ACT GTT CGA-3′). The PCR products (1,842 bp) were cloned into the TOPO-pCDNA2.0 vector (Invitrogen), which was digested with KpnI/NotI, and then the PCR products with the additional KpnI/NotI site were cloned into the pTracer CMV2 vector (Invitrogen), thus generating two plasmids, pTracer CMV2-CD24*^TG^* and pTracer CMV2-CD24*^del^* . The sequence of two CD24 cDNA inserts was confirmed by DNA sequencing. To exclude potential confounding factors, we selected the same sequence at the P170, P1056, and P1626 sites between the two plasmids.

### Cell culture and DNA transfection.

To test the expression efficiency of the *CD24* alleles, we transfected varying concentrations of plasmids into CHO cells using FuGENE 6 (Roche, http://www.roche.com). For the RNA stability experiment, 48 h after transfection, CHO cells were treated with actinomycin D (5 μg/ml) (Sigma, St. Louis, Mo.) for 0.5, 1, 2, 3, and 4 h. Untreated cells were used as control at the 0 h time point.

### RT-PCR.

We isolated total RNA from 1 × 10^6^ transfected CHO cells using a commercially available kit (Qiagen). We exposed RNA samples to DNase digestion before cDNA synthesis. For gene-specific PCR, 1 μl of first-strand cDNA product was amplified with platinum Taq polymerase (Invitrogen) according to the manufacturer's instructions. We designed primers specific for CD24 (forward: 5′-CCC ACG CAG ATT TAT TCC AGT-3′, reverse: 5′-TGG TGG TGG CAT TAG TTG GAT-3′) and for GFP (forward: 5′-GGT GAT GTT AAT GGG CAC AA-3′, reverse: 5′-TAG TGA CAA GTG TTG GCC ATG-3′) and performed a 30-cycle, three-step PCR (denaturation at 95 °C for 30 s, annealing at 55 °C for 30 s, extension at 68 °C for 30 s) with an initial temperature of 95 °C for 10 min.

An ABI Prism 7900-HT sequence system (Applied Biosystems, http://www.appliedbiosystems.com) with the QuantiTect SYBR Green PCR kit (Qiagen) was used in accordance with the manufacturer's instructions. A standard curve was created in each experiment using serial dilutions of positive template (plasmid). The relative amount of *CD24* mRNA was calculated by plotting the C*_t_* (cycle number) against the standard curve and comparing this to GFP as an endogenous control. The average relative expression for each group was determined using the comparative method (2^−ΔΔCt^). We used samples either without a template or with a template where the reverse-transcription step had been omitted as controls for unspecific contamination and amplification of plasmid DNA, respectively.

### Allele-specific mRNA expression assay.

Genomic DNA from eight OSU MS patients was initially genotyped to identify a P1527*^TG/del^* heterozygote and the corresponding cDNA samples were initially analyzed by using a TaqMan gene expression assay. Sample cDNAs were amplified in a model 7900-HT sequence system (Applied Biosystems) using the forward and reverse primers and a FAM dye-labeled TaqMan MGB probe with a TaqMan PCR reagent kit (Applied Biosystems). The sequence of primers and the probes for CD24 P1527*^TG/del^*, which were designed by Applied Biosystems, were 5′-AGAAGGCAAAATGTAAAGGAGTCAAACT-3′ for a forward P1527*^TG^* primer, 5′-FAM-TTCCAGTCTTCACTTCCC-TAMRA-3′ for a P1527*^TG^* probe, 5′-GTTGCTATTCTGATCCATAGTTGTTTTTTAAAGA-3′ for a reverse P1527*^TG^* primer, 5′-AGAAGGCAAAATGTAAAGGAGTCAAACT-3′ for a forward P1527*^del^* primer, 5′-FAM-AAGTGAAGACGAAGCTAATTT-TAMRA-3′ for a P1527*^del^* probe , and 5′-TTCTAAATGTTGCTATTCTGATCCATAGTTGT-3′ for a reverse P1527*^del^* primer. Quantitative PCR was carried out in 96-well optical reaction plates using a cDNA equivalent of 50 ng of total RNA for each sample in a volume of 50 ml using the TaqMan Universal PCR Master Mix (Applied Biosystems) according to the manufacturer's instructions. The mixed plasmid of pTracer CMV2-CD24*^TG^*and pTracer CMV2-CD24*^del^* in the same concentrations was used as a template for making the standard curve. The known concentrations of the serially diluted CD24 plasmid mix were employed as a standard for the quantification of the sample cDNAs. Each sample was assayed in triplicate and the intra-assay coefficient of variation was less than 1%. Experiments were repeated three times.

### Statistical analysis.

Case-control population study. Patients and normal controls were examined for any significant differences in their genotype (allele) distributions in each of the *CD24* polymorphisms at the population level. First, the Hardy-Weinberg equilibrium assumption was checked for each polymorphism for the cases and controls separately. After such data quality analyses, we performed χ^2^ tests for each polymorphism by comparing the distribution of the genotypes (alleles) of the cases to that of the normal controls. We computed the *p*-values of the test statistics using Monte Carlo simulations to avoid the assumptions of asymptotic distributions that may not be valid for small counts. Then, using the counts of one of the genotypes (allele) as a reference, the odds ratios for the remaining genotype (allele) variants were computed. The associated 95% confidence intervals for the odds ratios were obtained through bootstrap resampling methods. All Monte Carlo simulations were performed with 100,000 iterations.

### Analysis using family data.

Since case control studies are, in general, sensitive to population stratification, which may render the interpretation of results less than satisfactory, we also considered transmission disequilibrium tests using family data from three cohorts; the OSU MS samples, the MSGG MS samples, and Columbus Children's Hospital SLE samples. We checked for Mendelian inconsistencies and obligatory double recombination, using Merlin to test the typing errors.

The numbers of families with transmissions to affected offspring are: OSU MS, 87; MSGG, 63; and SLE cohort, 189. In these analyses, we hoped to confirm any significant association uncovered in the case control studies to further strengthen the results. Each polymorphism was examined for transmission disequilibrium using the TRANSMIT software [34,35] to uncover any association between the polymorphism and MS or SLE. TRANSMIT can deal with data from general pedigrees, the same type of data collected in our family studies.

### Linkage disequilibrium.

Analysis of the four SNPs was carried out using Haploview [49] software to study the LD pattern in the *CD24* gene using the Caucasian family data from the three independent cohorts. Specifically, pairwise LD measure *r^2^* was calculated for each of the six pairs of SNPs, and their results were displayed in a tilted matrix. While all families' data were used for phase (haplotype) information, only unrelated individuals were used for calculating the LD.

### Survival analysis.

For each SNP, we estimated the Kaplan-Meier survival curve for patients with each of the genotypes associated with the polymorphism. We calculated the observed survival time of a patient as the time from the first day of symptoms to reaching EDSS 6.0 (in years) or to the day of the last follow-up visit if EDSS 6.0 had not been reached. In the latter situation, the recorded survival time was treated as a censored observation. Association between the estimated survival curves and the underlying genotypes were then assessed using a log-rank test [50].

### Analysis of expression data.

A paired *t*-test was used to assess the effect of the dinucleotide deletion of P1527 on the allele-specific reduction of *CD24* mRNA. Due to the small sample size (eight individuals) used, the *p*-value was calculated based on 1,000,000 Monte Carlo simulations without making the normality assumption of the underlying population. For the mRNA stability data, an analysis of variance was performed to test the hypothesis that P1527 modulates *CD24* mRNA stability. The dependency through time was taken into account by modeling the covariance using an autoregressive process. To test our hypothesis, we contrasted the decay patterns of the *CD24^TG^* with those of the *CD24^del^*. In addition, we tested their difference at each individual time point.

## Supporting Information

Figure S1 *CD24* Sequence Based on Celera Database (31 KB DOC)

Figure S2 Comparison between Human *CD24* Genomic DNA Sequence, Based on Sequences of Five Clones of PCR Products from Each of Eight Individuals (Four Males and Four Females, a Total of 40 Clones), and That of NC_000024.8 from the NCBI Database (45 KB DOC)

Table S1 Distribution of *hCD24* Genotype Frequencies in Female and Male Control Participants is Inconsistent with Significant Contribution of Y-Chromosomal Genes (62 KB DOC)

Table S2 Characterization and Composition of Three Family Groups (52 KB DOC)

### Accession Numbers

The National Center for Biotechnology Information (NCBI) (http://www.ncbi.nlm.nih.gov) accession number for the putative *CD24* sequence on Chromosome Y is NC_000024.8.

The NCBI accession numbers for the *CD24* SNPs discussed in this paper are P1056 A/G, rs1058818; P1527 TG/del, rs3838646; and P1626 A/G, rs1058881.

## Abbreviations

CHO: Chinese hamster ovary
EDSS: expanded disability status scale
LD: linkage disequilibrium
MS: multiple sclerosis
MSGG: Multiple Sclerosis Genetics Group
OSU: Ohio State University
RFLP: restriction length fragment polymorphism
RT-PCR: real-time PCR
SLE: systemic lupus erythematosus
SNP: single-nucleotide polymorphism
