# Supplementary material for: A Dinucleotide Deletion in CD24 Confers Protection against Autoimmune Diseases
Source: PLoS Genet. 2007 Apr 6;3(4):e49. doi: 10.1371/journal.pgen.0030049 (PMC1847692; doi:10.1371/journal.pgen.0030049)
Supplement: Supplementary file 1 [file pgen.0030049.sg001.doc]

**Fig. S1.** *CD24* sequence based on Celara database. The position of the intron (black) and exons (blue) are indicated based on comparison to published cDNA sequence. The sequence used for the primer set for the first-round PCR is shown in pink, the three sets for the second-round PCR were shown in dark green, light green and yellow, respectively. The SNPs studied are highlighted in grey.

**(Exon1🡪)**

GGGTCTCGCCGGCTCGCCGCGCTCCCCACCTTGCCTGCGCCCGCCCGGAGCCAGCGGTTCTCCAAGCACCCAGCATCCTGCTAGACGCGCCGCGCACCGACGGAGGGGACATGGGCAGAGCAATGGTGGCCAGGCTCGGGCTGGGGCTGCTGCTGCTGGCACTGCTCCTACCCACGC

**(Intron 1🡪)**

NNNNNNNNNNNNNNNNNNNNNNNNNNNNNNNNNNNNNNNNNNNNNNNNNNNNNNNNNNNNNNNNNNNNNNNNNNNNNNNNNNNNNNNNNNNNNNNNNNNNNNNNNNNNNNNNNNNNNNNNNNNNNNNNNNNNNNNNNNNNNNNNNNNNNNNNNNNNNNNNNNNNNNNNNNNNNNNNNNNNNNNNNNNNNNNNNNNNNNNNNNNNNNNNNNNNNNNNNNNNNNNNNNNNNNNNNNNNNNNNNNNNNNNNNNNNNNNNNNNNNNNNNNNNNNNNNNNNNNNNNNNNNNNNNNNNNNNNNNNNNNNNNNNNNNNNNNNNNNNNNNNNNNNNNNNNNNNNNNNNNNNNNNNNNNNNNNNNNNNNNNNNNNNNNNNNNNNNNNNNNNNNNNNNNNNNNNNNNNNNNNNNNNNNNNNNNNNNNNNNNNNNNNNNNNNNNNNNNNNNNNNNNNNNNNNNNNNNNNNNNNNNNNNNNNNNNNNNNNNNNNNNNNNNNNNNNNNNNNNNNNNNNNNNNNNNNNNNNNNNNNNNNNNNNNNNNNNNNNNNNNNNNNNNNNNNNNNNNNNNNNNNNNNNNNNNNNNNNNNNNNNNNNNNNNNNNNNNNNNNNNNNNNNNNNNNNNNNNNNNNNNNNNNNNNNNNNNNNNNNNNNNNNNNNNNNNNNNNNNNNNNNNNNNNNNNNNNNNNNNNNNNNNNNNNNNNNNNNNNNNNNNNNNNNNNNNNNNNNNNNNNNNNNNNNNNNNNNNNNNNNNNNNNNNNNNNNNNNNNNNNNNNNNNNNNNNNNNNNNNNNNNNNNNNNNNNNNNNNNNNNNNNNNNNNNNNNNNNNNNNNNNNNNNNNNNNNNNNNNNNNNNNNNNNNNNNNNNNNNNNNNNNNNNNNNNNNNNNNNNNNNNNNNNNNNNNNNNNNNNNNNNNNNNNNNNNNNNNNNNNNNNNNNNNNNNNNNCTGTCCCTTCCCAGGCAGCCAGGTGACTACTTCCCACTTTCACCCAGAGGCCCGAGGTGCCTGCAGGGATTAGCGCCTGGAGAGCTCGGGCTCCCTGGGGACGTTGTCCCTGGGTCCCTGAGGACTCGCTTCCCCCTAGGAATGTCTTCGCGCTCCCCGCGCTCTACACAAAGAGAGGCGACGGTCCCTTTCGGGGAGGGCGGCTCACGCCCATCCTCCACCCTCGGAGTTGGATTGGGTATCCCGGAGAAGCTACGGCTATCTGGCCCTTTTTAAACAAACCCCGGTTCCGGGCTGAAGCCGAGGCGGGGACCCTCTCCTGACGCTGCGCTCTCTGCGAGGCAACCTTTCACGTCCATCGCTTGTCATCTTAGGATCCCCATGTTATTTTAGCCCAGATTGGCTAGTTCTGGGGAGGCAGCATGGTGGGACGATTCTGTCCCGAGTCCCCGCCAGGCTTTGTTCGGGTCGCCGCTACCAGCGCGGTCTCCCTAGATCCTCCATCCGGGGAACCTCGCCCCGGGTGCGGGTACCCGGGGCCGCGCAGCGCTGCCTCGAGGGTGTATGGATGCACCGCGCCGGCGAGAGAGACCGGGGGCTGGGCCTGGGAGACCCTAGCGGGGGCGGGGGCGAAGAAGGTACGTTTGTTTGGGTCAGTTCGGGTTTGGAGGATGGGAGTGACGGCTCTCCAGCGCCTTCGATTGGGGTTAATCGAGGACTGTCACTGGGGCTTCTCCCCCAAAGTTGGGCGCGCTGCGTCCTCCGTCCCCAGACATCGAAAGTCCGGGCTCACCTGGGAGTGCAGCCTGAGTGTGGAACGCGCTTCCCCACCGCTGGCGGGGTAACCGTACCCTATTGTCTTCCTCCCAGTCCGCTGCGCTCCCGAGCCTTCCTCAATTCTTTGATGGGGGAGGGGCGCGGCTGCGAGAAACCGGCCCCCTCCACTCCCCGCCCCATCTTCTCTCCGACCAGATTTTTTTCCTCCAACATTGTAAGTGAGAAGTTTTAATAATGCGAATAAATCTAGCTTGGGAGAGAAAATTTTGTTTAGTGTCAGTTTAATAAACATAATTTTTTTGTTGGTGTAGAGGATCTTTAGATCCTCAGATTGATACACCAGGTTTTAAAAAGAAAACATCACCTGCTGCAGTAAAACTCATTGCATGAATAAAGAACAGGTTACTTTGCGGGATCCCAAACAAGGTGTCCAAAACAGGCACCCTGTTTTAAGTAAATTACCATATTAATGCTTTTCTTTATTGGGAGTGCCGTTTCCTGATGGGACACACTAAATCTTGCACACAGTAGGTGCCCGATAAATAAATTTGACTTGATCAAGATTTTTTTGTTGTTGTCACTAATCTATTTTGGATAGGATTTGTACATTTCTGTAGTCAATATTTGTTTAATGACCTTATGGATTTTACAACAAAAGGAGCTGGTGCTGATGTAGGAGGGCTTTATCTTTGGATTAATAGGCAGCCAAATGTACACGGGGCCCAGACTCTTGCCTGGTGCCACGTCCTCCCTCCAGCCTGTAATCAGAAGCCCTTGTAAACCCTCCCTTGCAGTGAGGGCTGAAAACTCCGATCTGCCTTTGTTCCATGACAAAGGAGAGATTATCACGTGCCCTACTGGACTGTGCATTTCGTGGCTTGAAAAATCAGTGGTGAATGATGCCACTCAGACGCTGGGACACTGGGAGAACCTTGGTTCTTGGCCACAGCACTGAGCTTGGCGACCAGTGAGGCTGCTGCTGGTGAAACTTAACACCATAACCTCTCGTTGTCTTTTGTGAAATCGTTCCGACTTCATGGTGTTTTCTTGAAGTCATTCTGCCTTAAAGGAAAAACTCATTTTCTTTTTCAATTTAACACAAATATTGTGGCCATTTTTTAGAAGAGTTACTATTTCAAATTTCAAGGGAGAAGATAAGACTGCATTTCCCCCTTAAGGAAAAGGCTAAGTTACCAAGGCTGTATTAAATTGAGTATGTGAAAGTTACCTAGCAAATCCAGGATAGACAGTGACCCAATGAATTCTAGTTCCATAAGTCTCAGTTACAATATTTAAAAATCCCCATTTTTTTGTGCAACTTTAAAATCTCCCCCTTTCTGGGAAAATAGACCTTATTGCAATGCCATTAAGTCAATATAATCTTGGAAACTGAATTAGTTAACCTGGTCCTTTTTTCCTTTGCAAAGAAATAAAAACAGGAGTTATAGTTATTGAATAACTTTTTTGTTCTTGTTGCCACTTGGCATTTTTGAGGCAATCTCTAGGTAGTTTGTTTTGAAA **(Forward 1^st^ primer🡪)** CTAAAGAGAA TGACCTTGGTGGGTTGAGACAAGAATTCAGAAGTTAATGATGTTGGGTAAGAGAACAATGGTAAGAGAGCAATCTAAGAATATATCACCTACTTTAATTTTATATGAGAGTACGTGGAGGTAGCTGTGATGTGGAAATGTATCCATGTAACTTTTTATGACTGAATTAGTTAACCTGGTCCTTTTTTCCTTTGCAAAGAAATAAAAACA

**(Exon2🡪)**

AGATTTATTCCAGTGAAACAACAACTGGAACTTCAAGTAACTCCTCCCAGAGTACTTCCAACTCTGGGTTGGCCCCAAATCCAACTAATGCCACCACCAAGGC(/T)GGCTGGTGGTGCCCTGCAGTCAACAGCCAGTCTCTTCGTGGTCTCACTCTCTCTTCTGCATCTCTACTCTTAAGAGACTCAGGCCAAGAAACGTCTTCTAAATTTCCCCATCTTCTAAACCCAATCCAAATGGCGTCTGGAAGTCCAATGTGGCAAGGAAAAACAGGTCTTCATCGAATCTACTAATTCCACACCTTTTATTGACACAGAAAATGTTGAGAATCCCAAATTTGATTGATTTGAAGAACATGTGAGAGGTTTGACTAGATGATGGATGCCAATATTAAATCTGCTGGAGTTTCATGTACAAGATGAAGGAGAGGCAACATCCAAAATAGTTAAGACATGATTTCCTTGAATGTGGCTTGAGAAATATGGACACTTAATACTACCTTGAAAATAAGAATAGAAATAAAGGATGGGATTGTGGAATGGAGATTCAGTTTTCATTTGGTTCATTAATTCTATAAGGCCATAAAACAGGTAATATAAAAAGCTTCCATGATTCTATTTATATGTACATGAGAAGGAACTTCCAGGTGTTACTGTAATTCCTCAACGTATTGTTTCGACAGCACTAATTTAATGCCGATATACTCTAGATGAAGTTTTACATTGTTGAGCTATTGCTGTTCTCTTGGGAACTGAACTCACTTTCCTCCTGAGGCTTTGGATTTGACATTGCATTTGACCTTTTATGTAGTAATTGACATGTGCCAGGGCAATGATGAATGAGAATCTACCCCCAGATCCAAGCATCCTGAGCAACTCTTGATTATCCATATTGAGTCAAATGGTAGGCATTTCCTATCACCTGTTTCCATTCAACAAGAGCACTACATTCATTTAGCTAAACGGATTCCAAAGAGTAGAATTGCATTGACCA(/G)CGACTAATTTCAAAATGCTTTTTATTATTATTATTTTTTAGACAGTCTCACTTTGTCGCCCAGGCCGGAGTGCAGTGGTGCGATCTCAGATCAGTGTACCATTTGCCTCCCGGGCTCAAGCGATTCTCCTGCCTCAGCCTCCCAAGTAGCTGGGATTACAGGCACCTGCCACCATGCCCGGCTAATTTTTGTAATTTTAGTAGAGACAGGGTTTCACCATGTTGCCCAGGCTGGTTTCGAACTCCTGACCTCAGGTGATCCACCCGCCTCGGCCTCCCAAAGTGCTGGGATTACAGGCTTGAGCCCCCGCGCCCAGCCATCAAAATGCTTTTTATTTCTGCATATGTTGAATACTTTTTACAATTTAAAAAAATGATCTGTTTTGAAGGCAAAATTGCAAATCTTGAAATTAAGAAGGCAAAAATGTAAAGGAGTCAAAACTATAAATCAAGTATTTGGGAAGTGAAGACTG(/del)GAAGCTAATTTGCATTAAATTCACAAACTTTTATACTCTTTCTGTATATACATTTTTTTTCTTTAAAAAACAACTATGGATCAGAATAGCCACATTTA(/G)GAACACTTTTTGTTATCAGTCAATATTTTTAGATAGTTAGAACCTGGTCCTAAGCCTAAAAGTGGGCTTGATTCTGCAGTAAATCTTTTACAACTGCCTCGACACACATAAACCTTTTTAAAAATAGACACTCCCCGAAGTCTTTTGTTCGCATGGTCACACACTGATGCTTAGATGTTCCAGTAATCTAATATGGCCACAGTAGTCTTGATGACCAAAGTCCTTTTTTTCCATCTTTAGAAAACTACATGGGAACAAACAGATCGAACAGTTTTGAAGCTACTGTG**(🡨 reverse 2^nd^ primer)** TGTGTGAATGAACACTCTTGCTTTATTCCA GAATGCTGTACATCTATTTTGGATTGTATATTGTGTTTGTGTATTTACGCTTTGATTCATAGTAACTTCTTATGGAATTGATTTGCATTGAACACAAACTGTAAATAAAAAGAAATGGCTGAAAGAAAAAAAAAAAAAAGAATTCTGTGTTGCATATGTCCCAAATTTACTTTATCCAGTATATCATTGATGGGCATTTGGGTTGGTTCCAAGTCTTTGCTATTGTGAACAGTGCTGCAATAAATATACATGTGCACGTGTCTTTATAGTAGAATGATTTATATTCCTTTGGGTANNNNNNNNNNNNNNNNNNNNNNNNNNNNNNNNNNNNNNNNNNNNNNNNNNNNNNNNNNNNNNNNNNNNNNNNNNNNNNNNNNNNNNNNNNNNNNNNNNNNNNNNNNNNNNNNNNNNNNNNNNNNNNNNNNNNNNNNNNNNNNNNNNNNNNNNNNNNNNNNNNNNNNNNNNNNNNNNNNNNNNNNNNNNNNNNNNNNNNNNNN
