# Supplementary material for: A Dinucleotide Deletion in CD24 Confers Protection against Autoimmune Diseases
Source: PLoS Genet. 2007 Apr 6;3(4):e49. doi: 10.1371/journal.pgen.0030049 (PMC1847692; doi:10.1371/journal.pgen.0030049)
Supplement: Supplementary file 2 [file pgen.0030049.sg002.doc]

**Fig.S2.** Comparison between human CD24 genomic DNA sequence, based on sequences of 5 clones of PCR products from each of 8 individuals (4 males and 4 females, a total of 40 clones), and that of NC200004.8 from the NCBI database. The primers used to amplify the fragment were marked in pink. Differences in sequences between CD24 and NC200004.8 were marked in green. Three frequent variations found in at least two clones in an individual were considered SNP, marked in red. Note that while the primers used can potentially amplify the putative NC200004.8, none of the 40 clones sequenced matched the sequence, which suggests that the NC200004.8 annotation is unlikely correct.

```
hCD24  1	    CTAAAGAGAATGACCTTGGTGGGTTGAGCAAGAATTCAGAAGTTAATGATGTTGGGTAAG  60
             ||||||||||||||||||||||||||||||||||||||||||||||||||||||||||||
Y      1	    CTAAAGAGAATGACCTTGGTGGGTTGAGCAAGAATTCAGAAGTTAATGATGTTGGGTAAG  60

hCD24  61    AGAACAATGGTAAGAGAGCAATCTAAGAATATATCACCTACTTTAATTTTATATGAGAGT  120
             ||||||||||||||||||||||||||||||||||||||||||||||||||||||||||||
Y      61    AGAACAATGGTAAGAGAGCAATCTAAGAATATATCACCTACTTTAATTTTATATGAGAGT  120

hCD24  121   ACGTGGAGGTAGCTGTGATGTGGAAATGTATCCATGTAACTTTTTATGTATTTTAGATTT  180
             ||||||||||||||||||||||||||||||||||||||||||||||||||||||||||||
Y      121   ACGTGGAGGTAGCTGTGATGTGGAAATGTATCCATGTAACTTTTTATGTATTTTAGATTT  180

hCD24  181   ATTCCAGTGAAACAACAACTGGAACTTCAAGTAACTCCTCCCAGAGTACTTCCAACTCTG  240
             ||||||||||||||||||||||||||||||||||||||||||||||||||||||||||||
Y      181   ATTCCAGTGAAACAACAACTGGAACTTCAAGTAACTCCTCCCAGAGTACTTCCAACTCTG  240

hCD24  241   GGTTGGCCCCAAATCCAACTAATGCCACCACCAAGGCGGCTGGTGGTGCCCTGCAGTCAA  300 P170 C/T
             ||||||||||||||||||||||||||||||||||||||||||||||||||||||||||||
Y      241   GGTTGGCCCCAAATCCAACTAATGCCACCACCAAGGCGGCTGGTGGTGCCCTGCAGTCAA  300

hCD24  301   CAGCCAGTCTCTTCGTGGTCTCACTCTCTCTTCTGCATCTCTACTCTTAAGAGACTCAGG  360
             ||||||||||||||||||||||||||||||||||||||||||||||||||||||||||||
Y      301   CAGCCAGTCTCTTCGTGGTCTCACTCTCTCTTCTGCATCTCTACTCTTAAGAGACTCAGG  360

hCD24  361   CCAAGAAACGTCTTCTAAATTTCCCCATCTTCTAAACCCAATCCAAATGGCGTCTGGAAG  420
             ||||||||||||||||||||||||||||||||||||||||||||||||||||||||||||
Y      361   CCAAGAAACGTCTTCTAAATTTCCCCATCTTCTAAACCCAATCCAAATGGCGTCTGGAAG  420

hCD24  421   TCCAATGTGGCAAGGAAAAACAGGTCTTCATCGAATCTACTAATTCCACACCTTTTATTG  480
             ||||||||||||||||||||||||||||||||||||||||||||||||||||||||||||
Y      421   TCCAATGTGGCAAGGAAAAACAGGTCTTCATCGAATCTACTAATTCCACACCTTTTATTG  480

hCD24  481   ACACAGAAAATGTTGAGAATCCCAAATTTGATTGATTTGAAGAACATGTGAGAGGTTTGA  540
             ||||||||||||||||||||||||||||||||||||||||||||||||||||||||||||
Y      481   ACACAGAAAATGTTGAGAATCCCAAATTTGATTGATTTGAAGAACATGTGAGAGGTTTGA  540

hCD24  541   CTAGATGATGGATGCCAATATTAAATCTGCTGGAGTTTCATGTACAAGATGAAGGAGAGG  600
             ||||||||||||||||||||||||||||||||||||||||||||||||||||||||||||
Y      541   CTAGATGATGGATGCCAATATTAAATCTGCTGGAGTTTCATGTACAAGATGAAGGAGAGG  600

hCD24  601   CAACATCCAAAATAGTTAAGACATGATTTCCTTGAATGTGGCTTGAGAAATATGGACACT  660
             ||||||||||||||||||||||||||||||||||||||||||||||||||||||||||||
Y      601   CAACATCCAAAATAGTTAAGACATGATTTCCTTGAATGTGGCTTGAGAAATATGGACACT  660

hCD24  661   TAATACTACCTTGAAAATAAGAATAGAAATAAAGGATGGGATTGTGGAATGGAGATTCAG  720
             ||||||||||||||||||||||||||||||||||||||||||||||||||||||||||||
Y      661   TAATACTACCTTGAAAATAAGAATAGAAATAAAGGATGGGATTGTGGAATGGAGATTCAG  720

hCD24  721   TTTTCATTTGGTTCATTAATTCTATAAGGCCATAAAACAGGTAATATAAAAAGCTTCCAT  780
             ||||||||||||||||||||||||||||||||||||||||||||||||||||||||||||
Y      721   TTTTCATTTGGTTCATTAATTCTATAAGGCCATAAAACAGGTAATATAAAAAGCTTCCAT  780

hCD24  781   GATTCTATTTATATGTACATGAGAAGGAACTTCCAGGTGTTACTGTAATTCCTCAACGTA  840
             ||||||||||||||||||||||||||||||||||||||||||||||||||||||||||||
Y      781   GATTCTATTTATATGTACATGAGAAGGAACTTCCAGGTGTTACTGTAATTCCTCAACGTA  840

hCD24  841   TTGTTTCGACAGCACTAATTTAATGCCGATATACTCTAGATGAAGTTTTACATTGTTGAG  900
             ||||||||||||||||||||||||||||||||||||||||||||||||||||||||||||
Y      841   TTGTTTCGACAGCACTAATTTAATGCCGATATACTCTAGATGAAGTTTTACATTGTTGAG  900

hCD24  901   CTATTGCTGTTCTCTTGGGAACTGAACTCACTTTCCTCCTGAGGCTTTGGATTTGACATT  960
             ||||||||||||||||||||||||||||||||||||||||||||||||||||||||||||
Y      901   CTATTGCTGTTCTCTTGGGAACTGAACTCACTTTCCTCCTGAGGCTTTGGATTTGACATT  960

hCD24  961   GCATTTGACCTTTTATGTAGTAATTGACATGTGCCAGGGCAATGATGAATGAGAATCTAC  1020
             ||||||||||||||||||||||||||||||||||||||||||||||||||||||||||||
Y      961   GCATTTGACCTTTTATGTAGTAATTGACATGTGCCAGGGCAATGATGAATGAGAATCTAC  1020

hCD24  1021  CCCCAGATCCAAGCATCCTGAGCAACTCTTGATTATCCATATTGAGTCAAATGGTAGGCA  1080
             ||||||||||||||||||||||||||||||||||||||||||||||||||||||||||||
Y      1021  CCCCAGATCCAAGCATCCTGAGCAACTCTTGATTATCCATATTGAGTCAAATGGTAGGCA  1080

hCD24  1081  TTTCCTATCACCTGTTTCCATTCAACAAGAGCACTACATTCATTTAGCTAAACGGATTCC  1140
             ||||||||||||||||||||||||||||||||||||||||||||||||||||||||||||
Y      1081  TTTCCTATCACCTGTTTCCATTCAACAAGAGCACTACATTCATTTAGCTAAACGGATTCC  1140

hCD24  1141  AAAGAGTAGAATTGCATTGACCACGACTAATTTCAAAATGCTTTTTATTATTATTATTTT  1200 P1056 A/G
             |||||||||||||||||||||| |||||||||||||||||||||||||||||||||||||
Y      1141  AAAGAGTAGAATTGCATTGACCGCGACTAATTTCAAAATGCTTTTTATTATTATTATTTT  1200

hCD24  1201  TTAGACAGTCTCACTTTGTCGCCCAGGCCGGAGTGCAGTGGTGCGATCTCAGATCAGTGT  1260
             ||||||||||||||||||||||||||||||||||||||||||||||||||||||||||||
Y      1201  TTAGACAGTCTCACTTTGTCGCCCAGGCCGGAGTGCAGTGGTGCGATCTCAGATCAGTGT  1260

hCD24  1261  ACCATTTGCCTCCCGGGCTCAAGCGATTCTCCTGCCTCAGCCTCCCAAGTAGCTGGGATT  1320
             ||||||||||||||||||||||||||||||||||||||||||||||||||||||||||||
Y      1261  ACCATTTGCCTCCCGGGCTCAAGCGATTCTCCTGCCTCAGCCTCCCAAGTAGCTGGGATT  1320

hCD24  1321  ACAGGCACCTGCCACCATGCCCGGCTAATTTTTGTAATTTTAGTAGAGACAGGGTTTCAC  1380
             ||||||||||||||||||||||||||||||||||||||||||||||||||||||||||||
Y      1321  ACAGGCACCTGCCACCATGCCCGGCTAATTTTTGTAATTTTAGTAGAGACAGGGTTTCAC  1380

hCD24  1381  CATGTTGCCCAGGCTGGTTTCGAACTCCTGACCTCAGGTGATCCACCCGCCTCGGCCTCC  1440
             ||||||||||||||||||||||||||||||||||||||||||||||||||||||||||||
Y      1381  CATGTTGCCCAGGCTGGTTTCGAACTCCTGACCTCAGGTGATCCACCCGCCTCGGCCTCC  1440

hCD24  1441  CAAAGTGCTGGGATTACAGGCTTGAGCCCCCGCGCCCAGCCATCAAAATGCTTTTTATTT  1500
             ||||||||||||||||||||||||||||||||||||||||||||||||||||||||||||
Y      1441  CAAAGTGCTGGGATTACAGGCTTGAGCCCCCGCGCCCAGCCATCAAAATGCTTTTTATTT  1500

hCD24  1501  CTGCATATGTTGAATACTTTTTACAATTTAAAAAAATGATCTGTTTTGAAGGCAAAATTG  1560
             ||||||||||||||||||||||||||||||||||||||||||||||||||||||||||||
Y      1501  CTGCATATGTTGAATACTTTTTACAATTTAAAAAAATGATCTGTTTTGAAGGCAAAATTG  1560

hCD24  1561  CAAATCTTGAAATTAAGAAGGCAAAAATGTAAAGGAGTCAAAACTATAAATCAAGTATTT  1620
             ||||||||||||||||||||||||||||||||||||||||||||||||||||||||||||
Y      1561  CAAATCTTGAAATTAAGAAGGCAAAAATGTAAAGGAGTCAAAACTATAAATCAAGTATTT  1620

hCD24  1621  GGGAAGTGAAGACTGGAAGCTAATTTGCATTAAATTCACAAACTTTTATACTCTTTCTGT  1680 P1527 TG/del
             ||||||||||||||||||||||||||||||||||||||||||||||||||||||||||||
Y      1621  GGGAAGTGAAGACTGGAAGCTAATTTGCATTAAATTCACAAACTTTTATACTCTTTCTGT  1680

hCD24  1681  ATATACATTTTTTTTCTTTAAAAAACAACTATGGATCAGAATAGCCACATTTAGAACACT  1740 P1626 A/G
             |||||| ||||||||||||||||||||||||||||||||||||||||||||| ||| |||
Y      1681  ATATAC-TTTTTTTTCTTTAAAAAACAACTATGGATCAGAATAGCCACATTTGGAATACT  1739

hCD24  1741  TTTTGTTATCAGTCAATATTTTTAGATAGTTAGAACCTGGTCCTAAGCCTAAAAGTGGGC  1800
             ||||||||||||||||||||||||||||||||||||||||||||||||||||||||||||
Y      1740  TTTTGTTATCAGTCAATATTTTTAGATAGTTAGAACCTGGTCCTAAGCCTAAAAGTGGGC  1799

hCD24  1801  TTGATTCTGCAGTAAATCTTTTACAACTGCCTCGACACACATAAACCTTTTTAAAAATAG  1860
             ||||||||||||||||||||||||||||||||||| ||||| ||||||||||||||||||
Y      1800  TTGATTCTGCAGTAAATCTTTTACAACTGCCTCGAAACACAGAAACCTTTTTAAAAATAG  1859

hCD24  1861  ACACTCCCCGAAGTCTTTTGTTCGCATGGTCACACACTGATGCTTAGATGTTCCAGTAAT  1920
             ||||||||||||||||||||||||||||||||||||||||||||||||||||||||||||
Y      1860  ACACTCCCCGAAGTCTTTTGTTCGCATGGTCACACACTGATGCTTAGATGTTCCAGTAAT  1919

hCD24  1921  CTAATATGGCCACAGTAGTCTTGATGACCAAAGTCCTTTTTTTCCATCTTTAGAAAACTA  1980
             |||||||||||||||||||||||||||||||||||||||||||||||||||| |||||||
hCD24  1920  CTAATATGGCCACAGTAGTCTTGATGACCAAAGTCCTTTTTTTCCATCTTTACAAAACTA  1979

Y      1981  CATGGGAACAAACAGATCGAACAGTTTTGAAGCTACTGTG  2020
             |||| ||||||||||||||||| |||||||||||||||||
hCD24  1980  CATGAGAACAAACAGATCGAACCGTTTTGAAGCTACTGTG  2019
```
