# Supplementary material for: A Dinucleotide Deletion in CD24 Confers Protection against Autoimmune Diseases
Source: PLoS Genet. 2007 Apr 6;3(4):e49. doi: 10.1371/journal.pgen.0030049 (PMC1847692; doi:10.1371/journal.pgen.0030049)
Supplement: Supplementary file 3 [file pgen.0030049.st001.doc]

| **Table S1.** Distribution of *hCD24* genotype frequencies in female and male controls subjects is inconsistent with significant contribution of Y-chromosomal genes | | | | | | |  |
| --- | --- | --- | --- | --- | --- | --- | --- |
|  | **Female** |  |  | **Male** |  |  |  |
| **SNPs** | ***n*** | ***%*** |  | ***n*** | ***%*** | **χ***^2^* | ***p*** |
| **P1056** |  |  |  |  |  |  |  |
| ***AA*** | 146 | 27.4 |  | 56 | 31.1 |  |  |
| ***AG*** | 265 | 49.7 |  | 85 | 47.2 |  |  |
| ***GG*** | 122 | 22.9 |  | 39 | 21.7 | 0.906 | 0.636 |
| **HWE** *^b^* ***p* value** | 0.934 |  |  | 0.528 |  |  |  |
| **P1527** |  |  |  |  |  |  |  |
| ***TG/TG*** | 425 | 79.7 |  | 143 | 79.4 |  |  |
| ***TG/del*** | 99 | 18.6 |  | 35 | 19.4 |  |  |
| ***del/del*** | 9 | 1.7 |  | 2 | 1.1 |  |  |
| ***TG/del+del/del*** | 108 |  |  | 37 |  | 0.007 | 0.933 *^a^* |
| **HWE** *^b^* ***p* value** | 0.253 |  |  | 0.931 |  |  |  |
| **P1626** |  |  |  |  |  |  |  |
| ***AA*** | 364 | 68.3 |  | 120 | 66.7 |  |  |
| ***AG*** | 149 | 28.0 |  | 53 | 29.4 |  |  |
| ***GG*** | 20 | 3.8 |  | 7 | 3.9 | 0.164 | 0.921 |
| **HWE** *^b^* ***p* value** | 0.335 |  |  | 0.706 |  |  |  |
| *^a^* *TG/del* + *del/del* *vs*. *TG/TG* |  |  |  |  |  |  |  |
| *^b^* Hardy-Weinberg Equilibrium |  |  |  |  |  |  |  |
