# Supplementary material for: A Dinucleotide Deletion in CD24 Confers Protection against Autoimmune Diseases
Source: PLoS Genet. 2007 Apr 6;3(4):e49. doi: 10.1371/journal.pgen.0030049 (PMC1847692; doi:10.1371/journal.pgen.0030049)
Supplement: Supplementary file 4 [file pgen.0030049.st002.doc]

| **Table S2 Characterization and composition of three family groups** | | | | | |  |  |  |
| --- | --- | --- | --- | --- | --- | --- | --- | --- |
|  | **MSOSU family** | |  | **MSGG family** | |  | **SLE family** | |
|  | ***n*** | **%** |  | ***n*** | **%** |  | ***n*** | **%** |
| **Total pedigrees** | 101 |  |  | 49 |  |  | 187 |  |
| **Nuclear families** | 93 |  |  | 63 |  |  | 187 |  |
| **Parents** |  |  |  |  |  |  |  |  |
| **No parents** | 22 | 23.7 |  | 11 | 17.5 |  | 33 | 17.6 |
| **One parent** | 38 | 40.9 |  | 25 | 39.7 |  | 88 | 47.1 |
| **Both parents** | 33 | 35.5 |  | 27 | 42.9 |  | 66 | 35.3 |
| **Offspring** |  |  |  |  |  |  |  |  |
| **Female** |  |  |  |  |  |  |  |  |
| **Affected** | 80 | 64.0 |  | 82 | 57.3 |  | 171 | 66.0 |
| **Unaffected** | 45 | 36.0 |  | 61 | 42.7 |  | 88 | 34.0 |
| **Male** |  |  |  |  |  |  |  |  |
| **Affected** | 41 | 38.0 |  | 34 | 32.7 |  | 16 | 21.1 |
| **Unaffected** | 67 | 62.0 |  | 70 | 67.3 |  | 60 | 78.9 |
| SLE: Systemic Lupus Erythematosus | | |  |  |  |  |  |  |
| MSOSU: Multiple Sclerosis Ohio State University Group | | | | |  |  |  |  |
| MSGG: Multiple Sclerosis Genetics Group | | |  |  |  |  |  |  |
